# Syphilis Trends in the Central Savannah River Area (CSRA) of Georgia and South Carolina, USA

**DOI:** 10.3390/jcm7080190

**Published:** 2018-07-31

**Authors:** Rebecca B. Stone, Yunmi Chung, Benjamin E. Ansa

**Affiliations:** Institute of Public and Preventive Health, Augusta University, Augusta, GA 30912, USA; ychung@augusta.edu (Y.C.); bansa@augusta.edu (B.E.A.)

**Keywords:** syphilis, trends, Georgia, South Carolina, sexually transmitted infections, epidemiology, CSRA

## Abstract

There has been an alarming resurgence of early syphilis since 2000, especially in the southeast region, which has one of the highest rates of primary and secondary syphilis in the United States of America (USA). Although the Central Savannah River Area (CSRA) is the second most populous area in Georgia with a large presence of health care facilities, its counties have one of the lowest overall rankings in health outcomes. This study examined the syphilis rates and trends in the CSRA. Data from the Centers for Disease Control and Prevention (CDC) National Center for HIV/AIDS, Viral Hepatitis, STD, and TB Prevention’s AtlasPlus was used. Cases of primary and secondary syphilis diagnosed during 2010–2015 were analyzed to describe reported syphilis among CSRA residents. In the CSRA, between 2010 and 2015, the incidence rate of primary and secondary syphilis increased from 5.9 to 9.4 cases per 100,000 population. The lowest rate of syphilis was observed in 2011 (2.7 cases per 100,000) and the highest rate in 2015. In 2015, the highest syphilis rates were observed among males (15.9 per 100,000), non-Hispanic blacks (16.9 per 100,000), and persons between the ages 20–24 years (34.5 per 100,000). The relevance of preventive measures has been widely communicated, yet it is clear that risk-taking sexual behavior is on the rise. Greater effort is warranted to reduce risky behaviors that promote the transmission of syphilis, including areas outside of major metropolitan areas.

## 1. Introduction

In the last ten years, there has been a worldwide resurgence of early syphilis. To further compound this problem, concomitant infections with other sexually transmitted diseases (STD) such as HIV, gonorrhea, chlamydia, human papilloma virus (HPV), and other infections have been reported [1]. Such infections have potential for spread into the general population through mixed sexual networks and are most common among persons under age 30 [1]. Studies have shown that syphilis increases transmissions and acquisition of HIV infection and if left untreated is associated with significant long term complications negatively impacting the clinical course for those infected with HIV [2,3]. Overall, clinical management becomes complicated yielding higher medical costs for patients when infected with syphilis.

In 2014, the syphilis rates for the United States of America (USA) increased to 6.3 cases per 100,000 populations, the highest rate since 1994 as compared to the lowest rate of 2.1 cases per 100,000 population since 2000 and 2001 [1]. In 2015, syphilis cases rose to 7.5 per 100,000 representing a 19.0% increase from 2014 to 2015. The increased rate was reported in both women and men; however, the rise in cases was higher among men, particularly among bisexual and men who have sex with men (MSM) accounting for over 70% of the infections [1]. In 2014, national primary and secondary (P&S) syphilis rates for USA also increased in every age group 15–44 years of age and in every race and ethnicity group, except for Native Hawaiians. The highest rates of P&S syphilis were seen in black men aged 20–24 years and 25–29 years primarily in the West and South regions of the United States with a rate of 17.9% and 16.9% respectively. Rates of congenital syphilis cases also increased in every region of the country during 2013–2014 [1]. 

The Central Savannah River Area (CSRA) is made up of 13 Georgia and 8 South Carolina counties encompassing the Augusta metropolitan area, Georgia’s second largest metropolitan area. Served by two public health districts in this area, the CSRA is higher than the national average for rates of poverty (20.8% vs. 15.5% in 2014) and uninsured individuals (16.0% vs. 10.4% for persons under 65 years in 2014). Although the CSRA is the second most populous area in Georgia with a large presence of health care facilities, its counties have one of the lowest overall rankings in health outcomes [4]. The purpose of this study is to describe trends in syphilis over time in the Augusta, Georgia Metropolitan Area and compare those rates to the national level.

## 2. Methods

Data from the CDC National Center for HIV/AIDS, Viral Hepatitis, STD, and TB Prevention (NCHHSTP)’s AtlasPlus was used [5]. AtlasPlus is an online interactive tool that includes surveillance data on infectious diseases such as HIV, AIDS, viral hepatitis, sexually transmitted disease, and tuberculosis. The surveillance data is based on case reports of confirmed diagnoses obtained from state and local level notifiable disease reporting, national surveys, and other projects that monitor STD incidence and prevalence. 

The study sample included cases of primary and secondary syphilis reported between 2010 and 2015 in the Central Savannah River Area (CSRA), which is a region spanning across the border of Georgia (GA) and South Carolina (SC). The CSRA region includes twelve GA counties and seven SC counties: GA—Richmond, Columbia, Burke, McDuffie, Washington, Jefferson, Screven, Wilkes, Jenkins, Lincoln, Warren, and Glascock Counties; SC—Aiken, Edgefield, Barnwell, Saluda, Bamberg, McCormick, and Allendale Counties. The aggregated CSRA region data were obtained by submitting a data request with NCHHSTP and the national and state-level data were obtained directly from AtlasPlus. 

Available variables included, disease, year of diagnosis, reporting area, age group, race/ethnicity, and sex. Data were stratified by age category (15–19 years and 5 year intervals thereafter until an open ended interval at age 65 years or above), race/ethnicity, and sex. Incidence rate per 100,000 population was calculated for the CSRA region by age, race/ethnicity, sex categories, and overall. The population denominators used to compute these rates for the CSRA region were based on the U.S. Census Bureau population estimates, which were also obtained from NCHHSTP. Incidence rate was calculated by dividing the number of primary and secondary syphilis cases by the population for the corresponding calendar year and the resulting number was then multiplied by 100,000. Kendall’s Tau-b correlation test was used to test trend. The data analysis for this paper was generated using SAS software, Version 9.4 of the SAS System for Windows (SAS Institute Inc., Cary, NC, USA). Statistical significance was assessed at α = 0.05.

The data were de-identified and publically accessible, thus it was exempt from IRB review at Augusta University. 

## 3. Results

**Figure 1:**
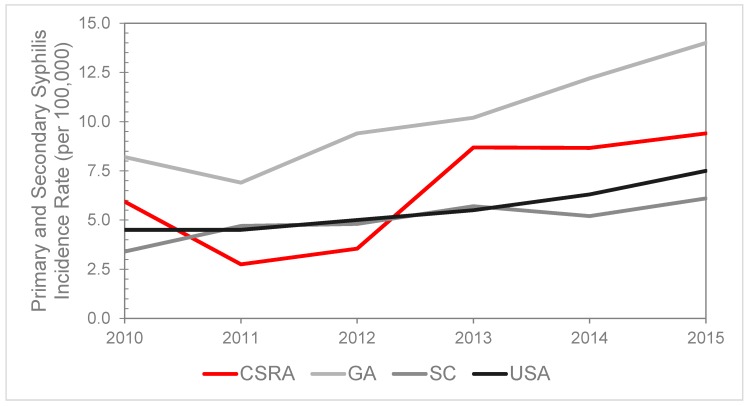
Incidence rate of primary and secondary syphilis in the Central Savannah River Area (CSRA) region compared to Georgia (GA), South Carolina (SC), and the national rates from 2010–2015.

In 2015, the national rate of primary and secondary (P&S) syphilis cases was 7.5 cases per 100,000 population. In the CSRA region, incidence rate of P&S syphilis was higher than the national rate at 9.4 per 100,000 population in 2015 (Figure 1). This was a 59.3% increase in incidence compared to 2010 (*p* = 0.056) and a 248.1% increase in incidence since 2011 (*p* = 0.023). The CSRA also had a higher rate in 2015 than observed SC (6.1 per 100,000 population), but lower than the rate for GA (14.0 per 100,000 population). The P&S syphilis rate fluctuated between 2010 and 2012, however, the rate increased every year since 2011. 

Overall in 2015, the highest rates of P&S cases were observed among 20–24 year olds, non-Hispanic blacks, and males in the CSRA (Table 1). The majority of the P&S syphilis cases were among men (82.9% in 2015). Between 2010 and 2015, the P&S syphilis rate fluctuated for women (between 1.1 and 3.2 cases per 100,000 CSRA females), however rates among men increased steadily since 2011. In 2015, the highest rates were observed among men between 20–24 years of age (59.5 cases per 100,000 CSRA males), followed closely by 25–29 year olds (47.9 cases per 100,000 CSRA males). Among black men, in 2015, the P&S syphilis incidence was the highest among 20–24 year olds at 121.8 cases per 100,000, which was almost eight times higher than white men of same age group (15.3 per 100,000). Such racial disparities in the P&S syphilis rates were observed for most age groups. 

## 4. Discussion

This study examined the rates and trends of P&S syphilis in the CSRA between 2010 and 2015 using data from the CDC National Center for HIV/AIDS, Viral Hepatitis, STD, and TB Prevention’s AtlasPlus. Incidence rate of P&S syphilis in the CSRA increased from 5.9 cases in 2010 to 9.4 cases per 100,000 population in 2015. CSRA had a higher rate in 2015 than observed nationwide and in SC, but lower than the rate for GA. However, P&S syphilis incidence rates in the CSRA were comparable to the overall P&S syphilis rate in the 50 most populous MSAs, which was 9.9 cases per 100,000 population [1]. Considering that Georgia ranked second among all 50 states in terms of highest P&S incidence rate, with a significant proportion of cases (77.6%) reported in the Atlanta metropolitan statistical area (MSA) [1], the rates observed in the CSRA are alarming. 

Rates of P&S syphilis continued to increase and racial and ethnic disparities have persisted throughout the USA [5]. The increases occurred primarily among black men. In 2013, the P&S syphilis rate among black men was 5.2 times that among white men (27.9 versus 5.4 cases per 100,000 population) and MSM accounted for the majority of male cases [6]. Also in 2013, highest rates of P&S diagnoses were observed among black men 20–29 years (between 96.4 and 97.2 cases per 100,000 men) [7]. These results are similar to those for the CSRA. Among black men in CSRA, in 2015, the P&S syphilis incidence was highest among 20–24 year olds at 121.8 cases per 100,000, which was almost 8 times higher than for white men of same age group (15.3 per 100,000). Since sexual orientation was not analyzed in the current study, we could not attribute the higher rate observed to MSM.

A plausible reason for the resurgence of P&S syphilis may be explained by increases in high-risk sexual behavior among people from all sociodemographic backgrounds [8]. There are diminished concerns about the risk of acquiring and transmitting STIs as a result of optimism regarding the availability of treatment for most of these conditions [8]. Also, the use of nitrate inhalants and other drugs, and an increased inclination to ignore messages promoting safe sex are thought to increase the risk for syphilis acquisition [8]. For example, a study that examined the use of methamphetamine, among MSM, reported an increase in the risk of syphilis acquisition [1,9,10,11]. Recently, the internet and social media have emerged as an important avenue for engaging sexual partners [12,13]. An association between the location of partners through the internet and acquisition of syphilis has been reported by a number of studies [14,15]. 

Syphilis and the risky behaviors associated with acquiring it increase the likelihood of acquiring and transmitting human immunodeficiency virus (HIV) [16]. In order to curb the increases in the incidence of syphilis, there is an urgent need for governments and policy makers to increase the awareness about the current epidemic and promote efforts to reduce high risk behaviors. Much of the messaging and reports have centered on large MSAs, which do evidently have the highest number of cases and incidence rates. However, as evidenced by the high rates in the CSRA, the communications and messaging should also expand beyond major MSAs. With the role that technology now plays even in people’s sex lives, more behavioral interventions that can reach a wider range of high-risk population (e.g., interventions/messaging potentially delivered through partnership with dating apps) are needed. Interventions that not only improve the skills of healthcare providers in diagnosing, screening and treating syphilis, but also behavioral interventions that educate patients are warranted. Specific prevention efforts to arrest the rise in syphilis resurgence in the CSRA should include the practice of safe sex through the use of condoms, dental dam, and avoiding sharing of sex toys. Educational interventions utilizing social media apps that target persons in the age range 20–24, with the highest incidence of syphilis should be promoted. Screening awareness programs that focus on education of patients by health care providers about the need to get tested for syphilis should be prioritized. Tested persons should be encouraged to discuss their test results with their partner. Since there has been a marked shift in the epidemiology of syphilis, with transmission primarily occurring among MSM, as opposed to heterosexual minority groups [17,18], Public Health prevention initiatives should also include programs for the MSM population.

This study has limitations. Firstly, we were limited by the number of sociodemographic variables that could be assessed. The study did not provide data on the incidence of P&S syphilis across levels of education and income, and sexual orientation of the study participants. Secondly, underestimating the true number of syphilis infections in P&S syphilis case-report data are likely because of underreporting of diagnosed cases. Finally, it would be interesting to investigate the rate of syphilis reinfection cases and sexual risk behaviors, but this information was not available in the dataset.

## 5. Conclusions

The relevance of preventive measures has been widely communicated, yet it is clear that risk-taking sexual behavior is on the rise as the United States continues to have increasing rates of syphilis. Funding for health care programs, such as STD clinics, have diminished over the past decade. The lack of funding has exposed vulnerable populations due to the lack of access to health care and resources necessary for screening and treatment. Greater effort is warranted to reduce risky behaviors that promote the transmission of syphilis and to gain a better understanding as to why rates for this disease continue to rise.

## Figures and Tables

**Table 1:** Incidence rate (per 100,000 population) of primary and secondary syphilis cases in the CSRA, United States, 2010–2015.

	2010		2011		2012		2013		2014		2015	
	*n*	Rate	*n*	Rate	*n*	Rate	*n*	Rate	*n*	Rate	*n*	Rate
Age in years												
15–19	6	11.2	4	7.9	1	2.0	2	4.1	6	12.6	3	6.3
20–24	11	22.4	5	9.8	12	22.8	15	28.3	16	30.1	18	34.5
25–29	4	8.28	2	4.1	5	10.1	8	16.0	17	33.3	13	24.8
30–34	5	11.3	5	11.0	3	6.4	10	21.0	7	14.6	12	24.9
35–39	3	6.8	0	0	2	4.7	11	25.8	3	7.0	7	15.8
40–44	0	0	0	0	1	2.2	6	13.2	3	6.7	3	6.8
45–54	9	8.5	4	3.8	0	0	10	9.9	8	8.1	9	9.2
55–64	5	5.5	0	0	1	1.1	1	1.0	2	2.1	5	5.0
65 +	0	0	0	0	1	1.0	0	0	1	0.9	0	0.0
Race/ethnicity												
White, non-Hispanic	6	1.5	4	1.0	5	1.2	15	3.7	12	3.0	20	4.9
Black, non-Hispanic	37	13.3	15	5.4	19	6.8	49	17.3	49	17.3	48	16.9
Asian/Pacific Islander	0	0	1	7.9	0	0	0	0	1	7.2	0	0.0
Am. Indian/Alaskan Native	0	0	0	0	0	0	0	0	0	0	0	0.0
Hispanic	0	0	0	0	2	5.9	0	0	2	5.5	1	2.7
Unknown	-	-	-	-	-	-	-	-	-	-	1	NA
Sex												
Male	36	10.2	14	3.94	22	6.13	46	12.7	55	15.2	58	15.9
Female	7	1.9	6	1.61	4	1.07	18	4.8	9	2.4	12	3.2
Total	43	5.9	20	2.7	26	3.5	64	8.7	64	8.7	70	9.4
